# Global contextual attention augmented YOLO with ConvMixer prediction heads for PCB surface defect detection

**DOI:** 10.1038/s41598-023-36854-2

**Published:** 2023-06-16

**Authors:** Kewen Xia, Zhongliang Lv, Kang Liu, Zhenyu Lu, Chuande Zhou, Hong Zhu, Xuanlin Chen

**Affiliations:** School of Mechanical and Power Engineering, Chongqing University of Science and Technology, Chongqing, 401331 China

**Keywords:** Computer science, Electrical and electronic engineering

## Abstract

To solve the problem of missed and false detection caused by the large number of tiny targets and complex background textures in a printed circuit board (PCB), we propose a global contextual attention augmented YOLO model with ConvMixer prediction heads (GCC-YOLO). In this study, we apply a high-resolution feature layer (P2) to gain more details and positional information of small targets. Moreover, in order to suppress the background noisy information and further enhance the feature extraction capability, a global contextual attention module (GC) is introduced in the backbone network and combined with a C3 module. Furthermore, in order to reduce the loss of shallow feature information due to the deepening of network layers, a bi-directional weighted feature pyramid (BiFPN) feature fusion structure is introduced. Finally, a ConvMixer module is introduced and combined with the C3 module to create a new prediction head, which improves the small target detection capability of the model while reducing the parameters. Test results on the PCB dataset show that GCC-YOLO improved the Precision, Recall, mAP@0.5, and mAP@0.5:0.95 by 0.2%, 1.8%, 0.5%, and 8.3%, respectively, compared to YOLOv5s; moreover, it has a smaller model volume and faster reasoning speed compared to other algorithms.

## Introduction

In applications such as the Internet of Things and artificial intelligence, PCB, as a key electronic device, plays a decisive role in the stability of the entire application^1^. To meet the current market demand for more sophisticated and complex electronic circuits, PCBs are moving toward precision, multiple layers, and miniaturization^2,3^. In the process of PCB manufacturing, circuit defects are one of the key factors leading to performance degradation. To ensure the quality of PCB products, it is necessary to achieve fast and efficient defect detection during PCB production.

There are many causes of PCB defects, such as soldering failure, manufacturing or storage conditions, and human factors. During PCB production, there are six common defects: missing hole, mouse bite, open circuit, short, spur, and spurious copper. This paper mainly studies these six defects. The characteristics of these defects are as follows: (1) PCB circuit designs are diverse, resulting in a complex background environment for PCB defects; (2) the defect area is small, and the color is similar to the background, which increases the difficulty of detection; and (3) while there are many kinds of defects, their characteristics are similar, which makes detection more difficult and prone to false detection and missed detection. These characteristics may lead to the following problems for PCB surface defect detection: (1) complex textures lead to the difficulty of the foreground and background distinction (2) tiny size leads to the problem of difficult detection. Currently, to address the above issues in visual detection, methods such as enhancing feature extraction, optimizing models, background modeling, data augmentation, multi-scale training, and attention mechanisms can be used to improve model performance. For the detection of complex background texture interference and tiny targets on the surface of PCB, the following development stages have been experienced.

Early PCB surface defect detection methods mainly include manual inspection, functional testing, and online testing. However, manual inspection is prone to visual fatigue, reducing detection efficiency and easily leading to misjudgment; the template cost of functional testing is high, and the detection cycle is long, which is not conducive to detecting various types of defects; and online testing only detects PCB electrical function defects, and thus, its detection scope and ability are limited. In recent years, PCB defect detection technology has developed, and a new detection method with automatic optical inspection as its core is becoming popular^4^. A PCB detection algorithm based on traditional imageology takes visual features such as texture, edge contour, and contrast as input to identify defects. However, these methods mainly relies on local features in the image when separating complex backgrounds, such as Scale-invariant feature transform (SIFT), Speeded Up Robust Feature (SURF), etc. Conversely, a PCB detection algorithm based on machine learning takes a large number of features obtained from visual image processing and classifies them through a classifier, thus achieving automatic detection of PCB surface defects. Machine learning methods such as support vector machines (SVMs)^5^, decision trees^6^, and random forests^7^ have been widely applied in PCB inspection, effectively improving the accuracy and efficiency of defect detection and enabling more types of defects to be detected. However, it still relies on effective feature information extraction algorithms and still struggles with the problems of complex foreground and background segmentation and small target detection on PCB surfaces.

In recent years, with the development and application of deep learning in various fields, several experts and scholars have focused on applying deep learning methods to the field of PCB surface defect detection. And by introducing new technologies such as candidate box generation^8^, feature pyramid networks^9^, and multi-scale detection^10^, it is possible to solve to some extent the difficulties encountered in detecting small targets and complex texture interference on PCB surfaces. Currently, deep learning-based object detection methods are mainly divided into one-stage methods and two-stage methods, according to the object localization manner. Two-stage object detection methods are mainly represented by Fast-RCNN^11^, Faster-RCNN^8^, and DetectoRS^12^ algorithms. Representative works of one-stage object detection mainly include YOLO series, SSD^13^, EfficientDet^14^, anchor-based RetinaNet^15^, CenterNet^16^, and anchor-free RepPoints^17^. Two-stage target detection algorithms have high recognition accuracy and are suitable for precise recognition scenarios; however, there are problems such as complicated training steps, slow detection speed, and large model volume. Thus, they are not suitable for PCB surface defect detection in industrial scenarios with lightweight and high-speed detection requirements. The one-stage algorithm eliminates tedious and time-consuming positioning operations and unifies the positioning and classification of detection objects using one network. It simplifies the network framework and training steps, improves the reasoning speed, and meets detection requirements in terms of detection accuracy and speed. In the one-stage target detection network, the YOLO series network has been widely used to various downstream tasks, and it has significant advantages over other models in terms of detection accuracy, speed, and deployment.

Therefore, this paper takes a one-stage object detection algorithm as the research direction, the YOLOv5s algorithm as a baseline model, research on methods such as multi-scale feature fusion, attention mechanism, and small target feature extraction and designs GCC-YOLO to solve the problems of high difficulty in distinguishing between PCB surface defects and background and small defect size leading to false detection and missed detection. The main contributions of this paper are given below.

1. We combine shallow high-resolution small target features and location information of the P2 layer with a BiFPN feature fusion path to better utilize shallow information, reduce shallow feature loss caused by the increase in model depth, and improve the model's detection ability for small targets.
2. We combine a GC module of the global context attention enhancement mechanism with a C3 module, thereby producing a new C3GC module. By enhancing the attention mechanism of the global context, this module can reduce the noise interference caused by shallow features and strengthen the feature extraction ability and anti-noise ability of the backbone network.
3. We introduce a ConvMixer module with large kernel depth separable convolution and pointwise convolution and combine it with the C3 module. A novel C3CM prediction head is designed to improve the receptive field of the prediction head and to enhance the model's ability to detect small targets while maintaining the model's light weight.

The rest of this paper is organized as follows. "Related work" section introduces related work on PCB surface defect detection. "Methodology" section details the proposed method in this article. "Experiment" section reports experimental data, experimental procedures, experimental results, and analysis of experimental results. Finally, in "Conclusion" section, we further summarize and analyze the proposed method in this article and consider further work.

## Related work

In this section, we first briefly review the related works of applying traditional visual algorithms, machine learning algorithms, and deep learning algorithms to PCB surface detection and analyze the advantages and disadvantages of each stage of PCB automatic detection. Second, we introduce the detection performance of the model proposed in this paper.

In the research on traditional visual inspection algorithms for PCB detection, Gaidhane et al.^18^ proposed an efficient similarity measurement method for detecting and localizing local defects in complex component-mounted PCB images. The proposed method is effective in detecting and locating local defects in complex component-mounted PCB images. Tsai et al.^19^ proposed a global Fourier image reconstruction method to detect and locate PCB surface defects, and it could adapt to translation and illumination transformation environments. Abdel-Aziz Hassanin et al.^20^ proposed a real-time PCB automatic defect detection method based on SURF features and morphological operations, and it could accurately determine the fault location and fault type. Although traditional detection algorithms based on image processing have certain detection effects, this type of detection method often has strict application conditions and cannot meet industrial needs in terms of robustness and real-time performance.

In the research on machine learning-based PCB detection algorithms, Tsai et al.^21^ proposed two entropy measures, i.e., color and structure, and input them to an SVM classifier for automatic defect detection of PCB edge connectors. The proposed method was effective and efficient for detecting defects such as pinholes, copper exposure, scratches, and roughness on gold-plated surfaces. Lu et al.^22^ proposed a PCB defect detection framework based on Bayesian feature fusion; it achieved good results in terms of speed and accuracy, even for scenes such as those with uneven lighting and camera angle tilt. Thanasis Vafeiadis et al.^23^ proposed a framework for detecting PCB surface defects and inferring failures of excessive or insufficient glue, with SVM as the optimal classifier. Although target detection algorithms based on machine learning have overcome the shortcoming of poor robustness of traditional detection algorithms to a certain extent, they still face problems such as large data volume, redundant information, and high-dimensional feature space. Moreover, they are easily affected by factors such as environment, light, production process, and noise.

Regarding research on PCB detection algorithms based on a two-stage object detection network, Hu et al.^24^ proposed an improved Faster RCNN algorithm based on the feature pyramid network ResNet50, thus improving the detection ability of small defects in PCBs. Meanwhile, Zeng et al.^25^ proposed an enhanced multi-scale feature fusion algorithm based on the asymmetric balanced feature pyramid network (ABFPN) and realized effective small target detection. Zhang et al.^26^ proposed the Cost-Sensitive Residual Convolutional Neural Network (CS-ResNet), which effectively balances the different misclassification costs of sample imbalance and real and false defects in PCB detection. Two-stage object detection algorithms have high recognition accuracy and are suitable for precise recognition scenarios but face problems such as complicated training steps, slow detection speed, and large model volume. Therefore, they are not suitable for PCB surface defect detection in industrial scenarios with lightweight and high-speed detection requirements.

To solve the problems of two-stage object detection methods, researchers proposed one-stage object detection algorithms, which eliminate the time-consuming localization operations and unify the localization and classification of the detection objects into one network, thus simplifying the network framework and training steps and improving inference speed. For instance, Kang et al.^27^ proposed an improved multi-layer SSD algorithm that addresses the false detection problem in PCB defect detection to some extent. Chen et al.^28^ proposed an improved EfficientDet algorithm with parallel convolution modules, serial convolution modules, and feature fusion modules and achieved significant performance in detecting small defects. Bhattacharya et al.^29^ proposed an improved YOLOv5 algorithm based on a Transformer backbone, which reduced the parameters of YOLOv5 while maintaining good detection accuracy. Overall, the one-stage object detection algorithm maintains a fast detection speed and good detection accuracy but performs poorly in high-precision small target detection.

Based on the problems of the above algorithm, in order to improve the accuracy of PCB surface defect recognition and detection speed while meeting the requirements of lightweight deployment, this paper takes a one-stage object detection algorithm as the research direction, the YOLOv5s algorithm as a baseline model, and proposes an improved GCC-YOLO algorithm. The proposed algorithm deals with a large number of small targets and complex background noise interference on the PCB surface. This algorithm achieves 98.2% mAP@0.5, 76.7% mAP@0.5:0.95, 68.0 FPS, and 14.5 Mb model size on the PCB dataset, which verifies its effectiveness.

## Methodology

To address the small size of surface defects on PCB, high similarity between defects and the background, and low distinction between different types of defects, this paper proposes a global context attention enhancement and improved ConvMixer prediction head YOLO (GCC-YOLO) based on YOLOv5s. In this section, the basic framework of YOLOv5s is introduced first. This is followed by a detailed description of the overall framework and four specific improvements brought by the proposed method.

### Review of YOLOv5

YOLO is a series of object detection algorithms based on deep learning and convolutional neural network. There are four models of YOLOv5, i.e., YOLOv5s, YOLOv5m, YOLOv5l, and YOLOv5x, with increasing depth and width of the network in that order. This paper chooses YOLOv5s as a baseline model and uses the pre-trained weights based on the COCO dataset for transfer learning to establish a PCB surface defect detection algorithm.

The YOLOv5s model consists of four main parts: the input, backbone, neck, and head. The input network uses Mosaic data augmentation, randomly combining four images each time to increase the diversity of the dataset. The backbone network is used to extract image features and consists mainly of a C3 module and an SPP module. The C3 module is composed of a bottle-neck structure and convolution layers, which accelerates the inference process. The SPP module uses three pooling kernels with sizes of 5, 9, and 13 to perform maximum pooling on the input image, which greatly enhances the receptive field of the network. The neck is the fusion part of the network, and it adopts a combination of feature pyramid networks (FPNs)^9^ and pyramid attention networks (PANs)^30^. It can effectively enhance the fusion effect of features of different scales. Finally, the head is the prediction part of the network, and it outputs three vectors containing the predicted box class, confidence, and coordinate location.

### Improved network architecture

**Figure 1:**
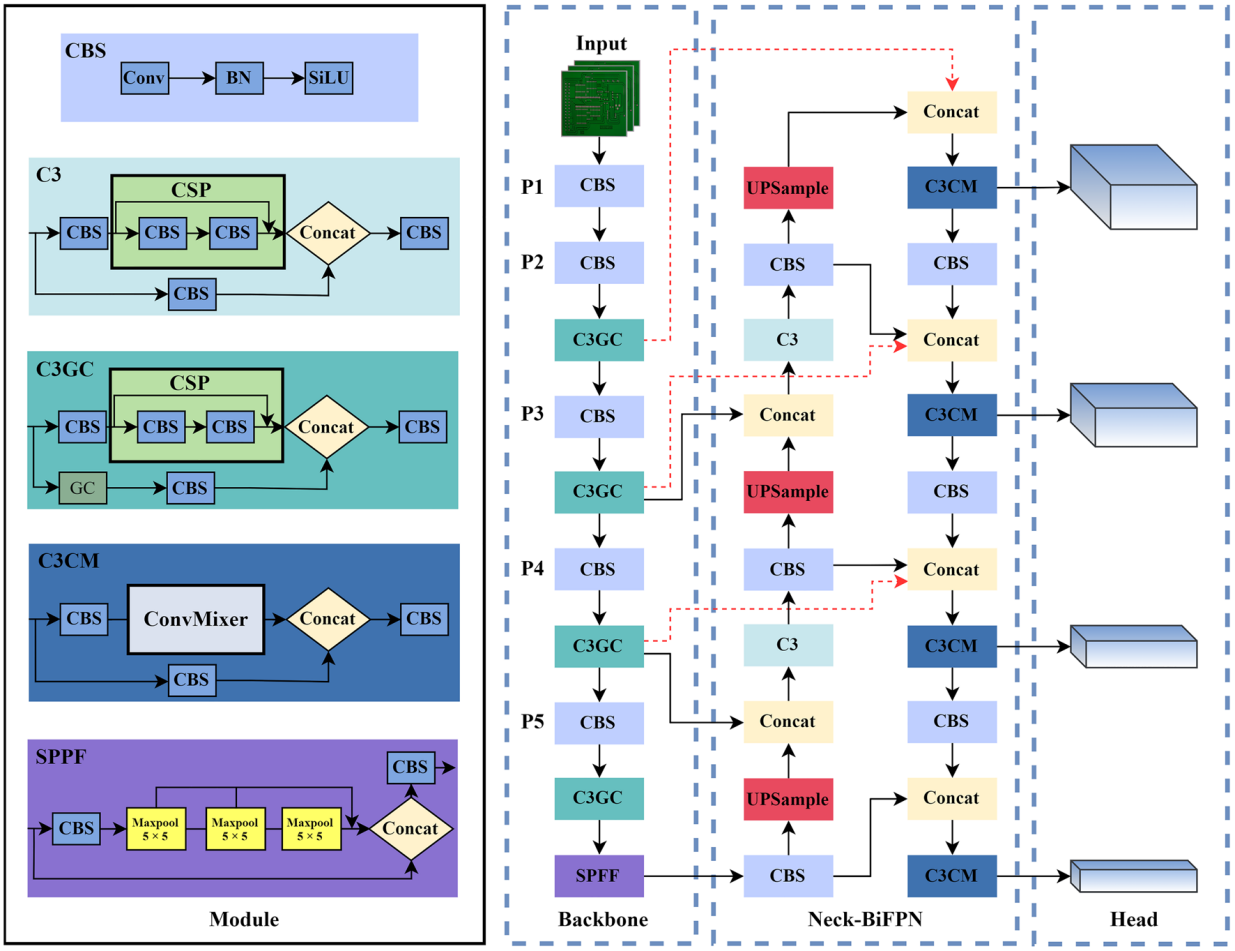
Structure of GCC-YOLO.

The framework of the GCC-YOLO proposed in this paper is shown in Fig. 1. The main improvements of GCC-YOLO are as follows. (1) To fully utilize the position and detail information of small targets provided by the shallow feature layer, a new feature fusion layer and a prediction head are added to the neck network to enhance the detection ability of small targets and make the network adapt to scale changes of different types of defects on the PCB surface; (2) To extract more effective feature information, a global context attention enhancement (GC) module is introduced and combined with a C3 module, thus producing a new C3GC module. By making full use of global context information, this module can improve the ability to distinguish between defect areas and complex backgrounds, reduce noise interference caused by shallow features, and enhance the feature extraction and anti-noise capabilities of the backbone network; (3) We introduce a bidirectional weighted feature pyramid (BiFPN) feature fusion structure in the neck network to reduce the loss of shallow information and improve model accuracy; (4) We introduce a ConvMixer module with large kernel depth separable convolution and pointwise convolution and combine it with the C3 module. A novel C3CM prediction head is designed to improve the receptive field of the prediction head and to enhance the model's ability to detect small targets while maintaining the model's light weight.

### High-resolution feature layer P2

The existing small target definition methods are mainly divided into the following two categories: relative scale-based definitions and absolute scale-based definitions. In a study on relative scale-based definitions, Chen et al.^31^ proposed that the median of the ratio of the area of the bounding box to the area of the image in the same category is between 0.08 and 0.58%, which is considered as small targets. In a study based on the absolute scale definition, Torralba et al.^32^ defined small targets as targets with a resolution of less than 32 × 32 pixels. When extracting features in convolutional neural networks, due to the small pixel ratio of the target object, the feature information will gradually decrease after several down-sampling layers, and will continue to be lost as the network level deepens^33^.

**Figure 2:**
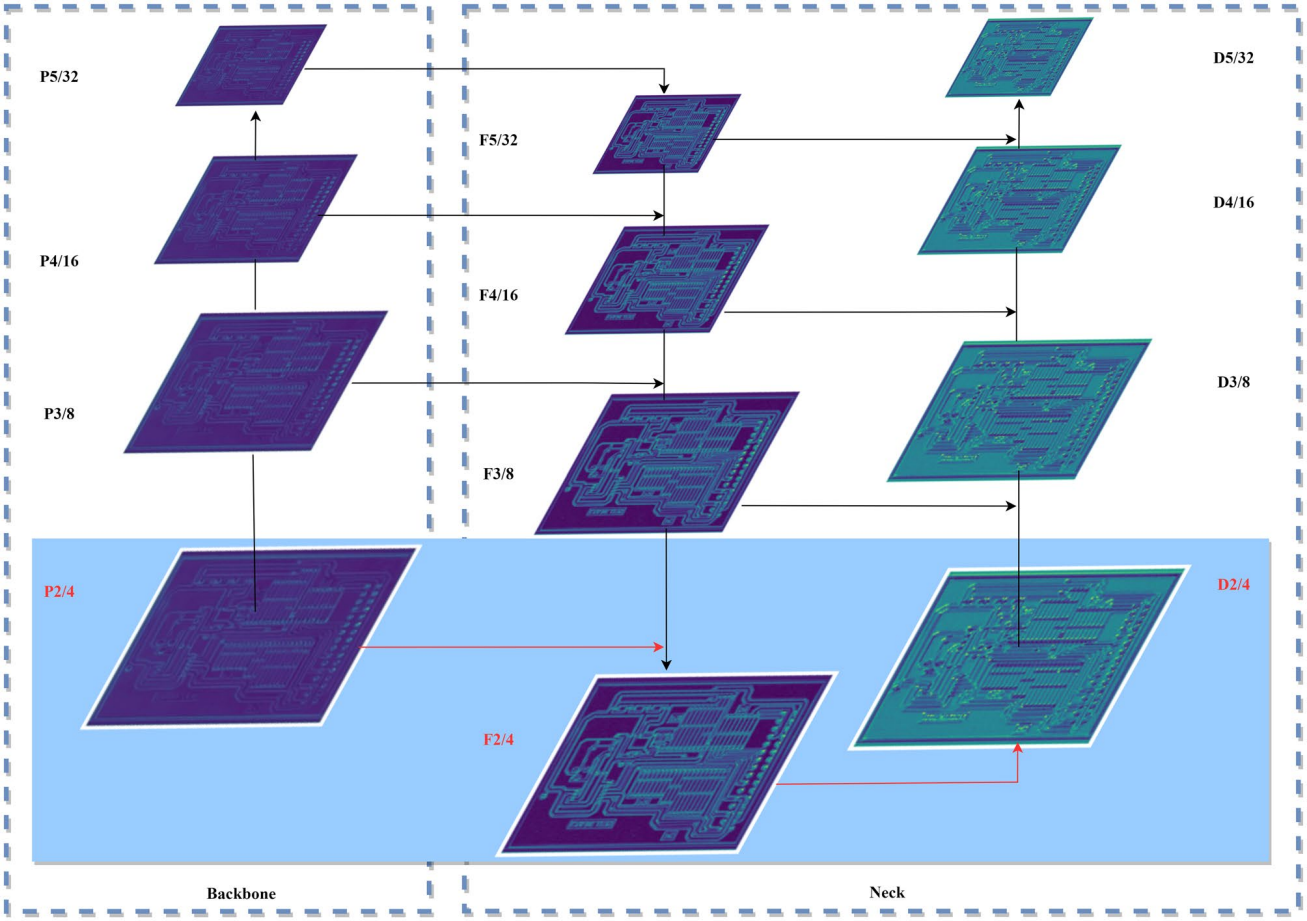
Structure with high-resolution detection layer P2.

The backbone network of YOLOv5s adopts an FPN structure, which continuously extracts features through three down-sampling layers and outputs down-sampled feature maps p3, p4, and p5 of 1/8, 1/16, and 1/32 sizes of the original image, respectively. A 1/8 size version of the P3 feature map is used as input to the neck network for feature fusion for small target detection. However, there are a large number of small targets in the PCB dataset, as shown in Table 1. We notice that there is a certain proportion of tiny targets with a size less than 10 × 10 pixels, for which the target features become very weak or even disappear completely in the feature map after 1/8 down-sampling. Therefore, a shallow high-resolution feature map P2 with 1/4 down-sampling is introduced into the backbone network, as shown in Fig. 2. In response, an F2 layer and a D2 layer are added to the neck network for feature fusion and micro target prediction, respectively.

**Table 1:** Distribution of small targets in the PCB dataset.

Type	Number		Area (pix)		
	Image	Instance	≤ 10 × 10	≤ 16 × 16	≤ 32 × 32
Missing hole	690	2889	2	962	1875
Open circuit	690	2804	380	1740	682
Mouse bite	696	2886	92	1255	1508
Short	696	2868	11	384	2209
Spur	690	2863	36	672	2095
Spurious copper	696	2947	10	485	2389

### Enhanced backbone with global contextual attention

**Figure 3:**
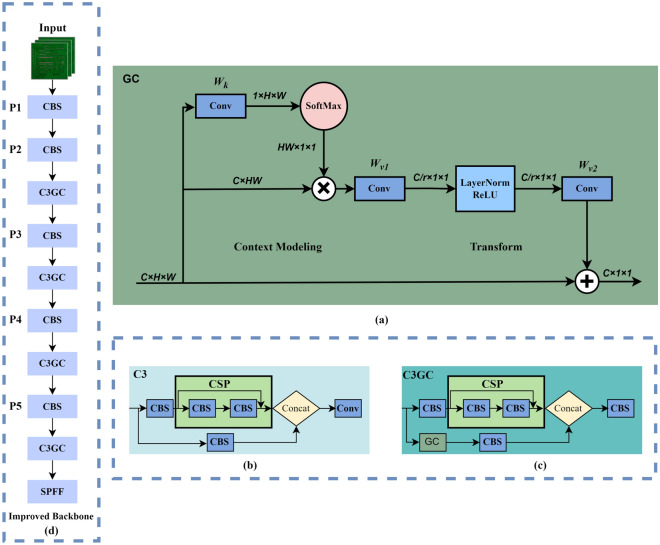
Illustration of the backbone with global contextual attention: (**a**) global contextual attention backbone structure, (**b**) C3 module with residual structure, (**c**) proposed C3GC module with global contextual attention, and (**d**) Enhanced Backbone with Global Contextual Attention.

Due to the high similarity between target defects and the background in the PCB dataset, noise interference is common. Moreover, the introduced shallow feature map also carries a large amount of background information, if it is directly transmitted to the neck network without effective processing, it will inevitably cause the feature information of small targets to be overwhelmed by background noise, which in turn affects the detection accuracy of small targets. For the above problems, we notice that strengthening the extraction of global context information can simultaneously suppress background noise and enhance the feature information extraction ability of small targets. Therefore, we introduce a global contextual attention module (GC)^34^, as shown in Fig. 3a. The GC module consists of context modeling, a bottleneck transform, and broadcast element-wise addition.

In the backbone network, the C3 module contains two feature propagation paths (see Fig. 3b). The first feature propagation path is fed into the CSP Block^35^ containing the residual connections after a 1 × 1 convolution, while the second feature propagation path fuses the features of these two feature propagation paths after a 1 × 1 convolution to process the number of channels. However, in the second feature propagation path, there is a lot of unprocessed original information, which will lead to a lot of noise interference during feature fusion. Therefore, the GC module is introduced in the second feature processing path to build a C3GC module with global context attention enhancement, which can well solve the problems of background noise interference and small target information loss. The C3GC module is shown in Fig. 3c. Replacing all C3 modules in the backbone network with C3GC modules results in the model retaining more small target feature details during each down-sampling (see Fig. 3d). This also, to some extent, weakens the shallow noise interference brought by the P2 layer, which is beneficial for the detection of tiny defects in PCB.

### Feature fusion

**Figure 4:**
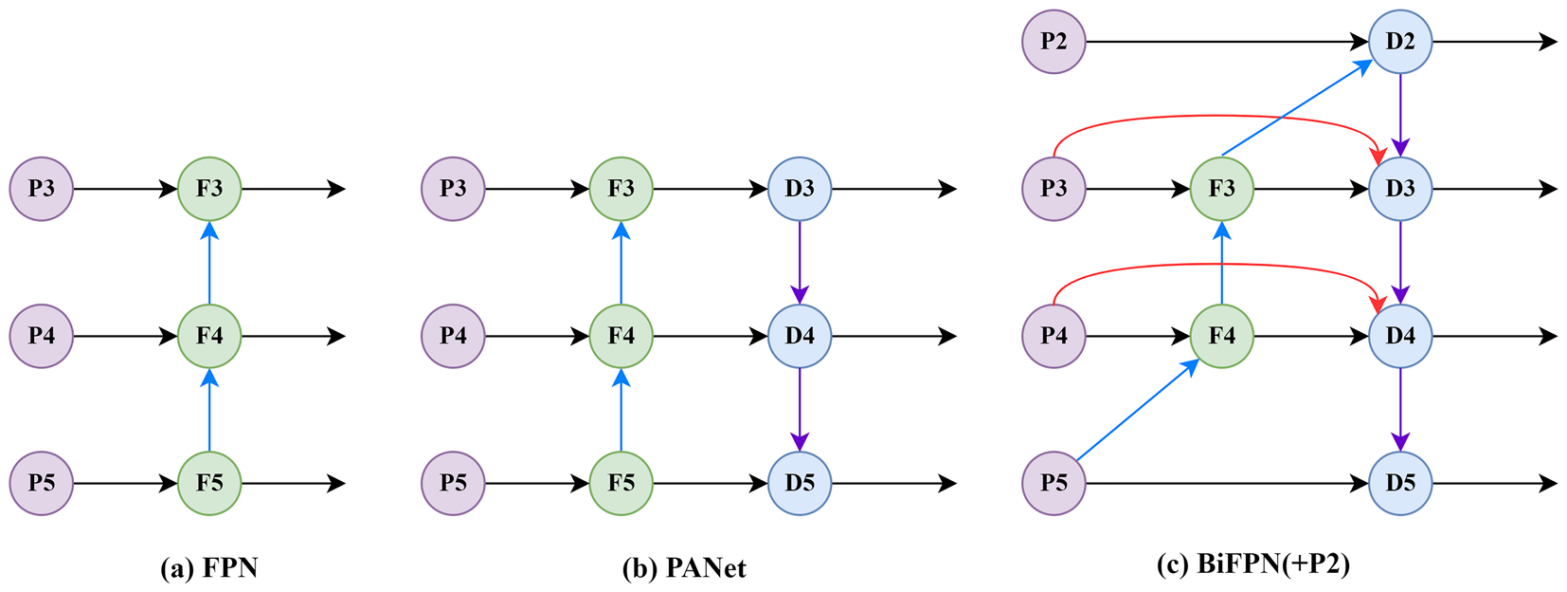
Feature network design: (**a**) FPN, (**b**) PANet, and (**c**) BiFPN.

YOLOv5s’ neck network uses a combined structure of an FPN and PAN and fuses the features of shallow feature layers and deep feature layers (see Fig. 4b). The FPN can transfer deep semantic information to shallow layers without affecting the positional information, while the PAN can transfer the positional information contained in the shallow feature layer to the deep feature layer. This combination effectively improves accuracy in network feature fusion, but it also brings some problems. All the inputs of the PAN are feature information processed by the FPN and thus lack the original feature information of the trunk feature extraction network; this makes it easy to cause bias in training and learning and thus affects the accuracy of detection. EfficientDet^15^ uses a BiFPN feature fusion structure that effectively fuses shallow feature information of the backbone network and can well solve the problem of shallow feature loss. Hence, we replace the PANet feature fusion path in the original network architecture with BiFPN and remodel the feature fusion path of YOLOv5s neck network, as shown in Fig. 4, for FPN, PANet, and BiFPN structures.

### ConvMixer prediction head

**Figure 5:**
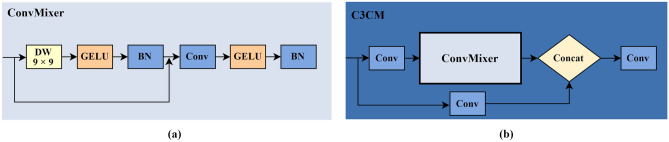
Illustration of the C3CM module: (**a**) ConvMixer Layer, (**b**) C3CM module.

In YOLOv5s, the prediction head adopts a C3 module as the general object detection head; but this is not specifically designed for small targets. Following its widespread application in the field of computer vision, the Transformer^36^ algorithm has become another mainstream architecture after the RNN and CNN. Studying efficient attention architectures based on Transformer, Xingkui Zhu et al.^37^ proposed a TPH-YOLO algorithm by introducing Transformer as the prediction head; they achieved high accuracy on small target detection on a drone target detection set, proving that the prediction head of YOLOv5s has shortcomings in detecting small targets. In subsequent studies, ConvMixer^38^ achieved performance comparable to Vision Transformer with fewer resources by mixing data spatially and channel-wise. This paper introduces a ConvMixer architecture as a new prediction head to improve the detection of small targets. In ConvMixer, the ConvMixer Layer is its core component, as shown in Fig. 5a. It consists of depthwise separable convolution, pointwise convolution, a GELU activation function, and a BN, with a residual connection in one of the groups. The depthwise separable convolution uses a 9 × 9 kernel design to ensure a larger field of view, and the pointwise convolution can enhance the detection of tiny targets. We combine the ConvMixer Layer with the C3 module to replace the CSP structure in the original C3 module and thus form a new C3CM module to replace the prediction head in YOLOv5s (see Fig. 5b). The new detection head brings a larger field of view to improve the model's detection and recognition capabilities.

## Experiment

### Experimental data

**Figure 6:**
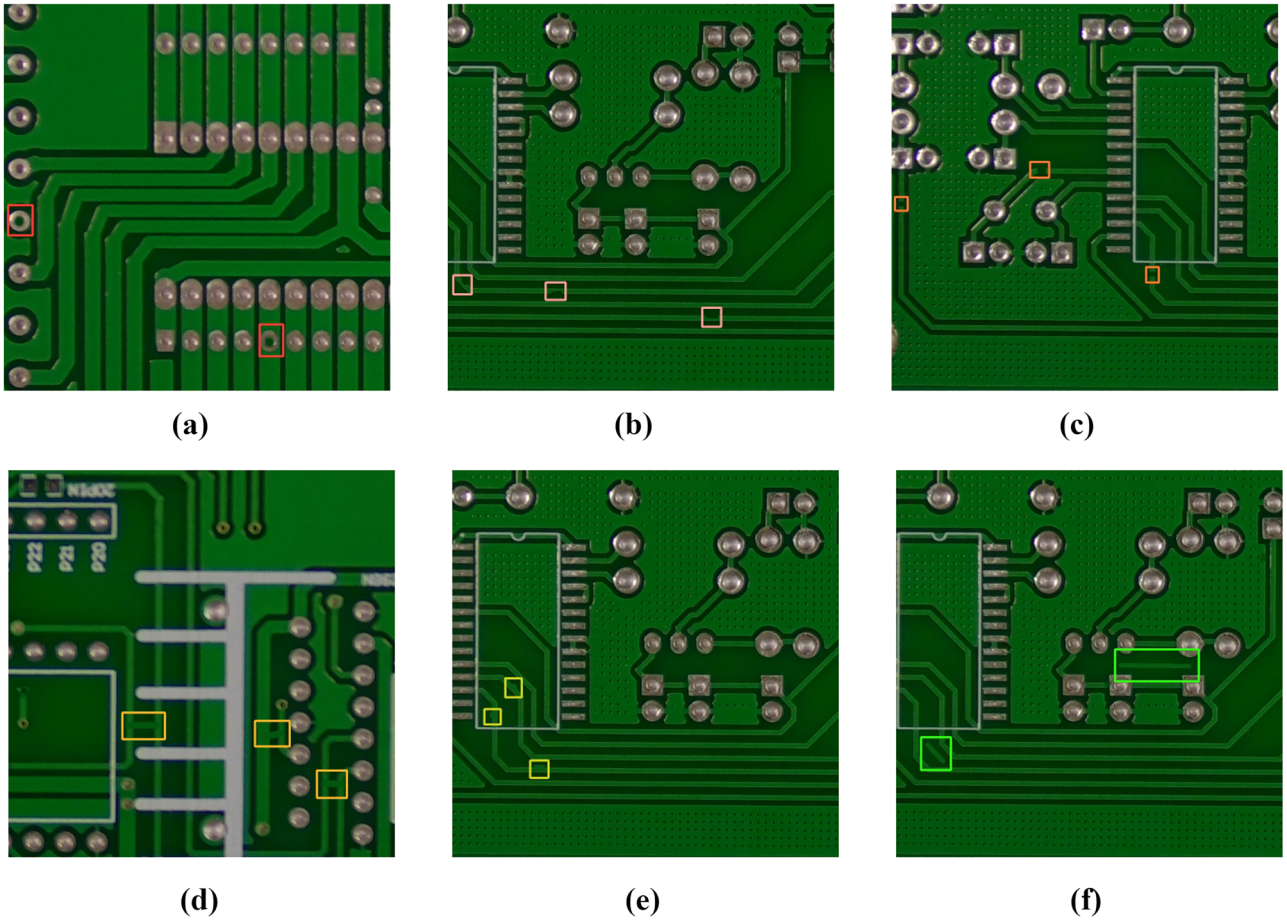
Six different kinds of defects: (**a**) Missing hole, (**b**) Mouse bite, (**c**) Open circuit, (**d**) Short, (**e**) Spur, and (**f**) Spurious copper.

**Figure 7:**
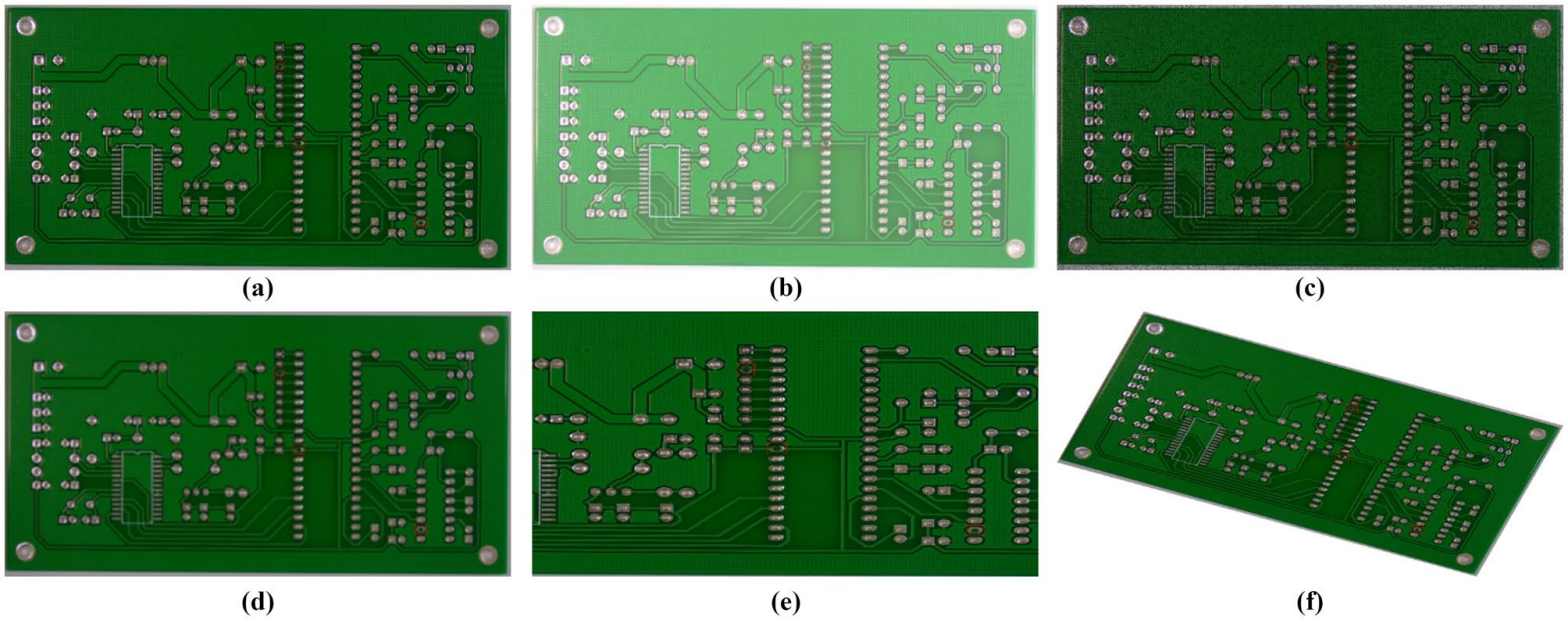
Example of Image Enhancement: (**a**) Original image, (**b**) Brightness adjustment, (**c**) Add noise, (**d**) Motion blur, (**e**) Randomly zoom, and (**f**) Random rotation.

The dataset adopted in our experiment is the PCB defect dataset^39^ released by the Open Lab of Peking University; its defect types include missing hole (Mh), mouse bite (Mb), open circuit (Oc), short (Sh), spur (Sp), and spurious copper (Sc), and it contains a total of 693 images. Examples of the six types of defects are shown in Fig. 6. To restore complex situations that may occur during automatic PCB inspection, the original dataset is enhanced through image enhancement algorithms such as motion blur, random zooming, cropping, random light and dark, salt and pepper noise, and random rotation. An enhanced image is shown in Fig. 7. After enhancement, the dataset is expanded to 4158 images. The distribution of various defects and instances in the dataset is shown in Table 1. The training set, validation set, and test set are divided in the ratio of 8:1:1, with 3326 images in the training set and 416 images in both the test set and validation set.

### Experimental environment and training parameters

This experiment is built on an AutoDL server, with an RTX3090 GPU, Intel(R)Xeon(R)Platinum8358P CPU, running on a Linux operating system, using PyTorch1.8.1, Python3.8, and CUDA11. The experimental environment was set up as shown in Table 2.

**Table 2:** Experimental environment.

Experimental environment	
Processor	Intel(R)Xeon(R)Platinum8358P CPU
Operating system	Linux
Ram	32 GB
Graphics card	RTX3090 GPU
Programming language	Python3.8
Deep learning libraries	PyTorch1.8.1
Deep learning toolkit	CUDA11

To train our models, the Stochastic Gradient Descent (SGD) optimizer was used with a momentum and weight decay of 0.937 and 0.0005, respectively. And the learning rate was adjusted as 0.01. To find the optimal hyperparameter values, we chose image size 640 × 640, batch size 32, and the model was run for up to 300 epochs. The training parameters are shown in Table 3.

**Table 3:** Training parameters.

Parameter	Value
Image Size	640*640
Learning Rate	0.01
Weight Decay	0.0005
Momentum	0.937
Optimizer	SGD
Batch Size	32
Dataloader	16
Epoch	300

**Figure 8:**
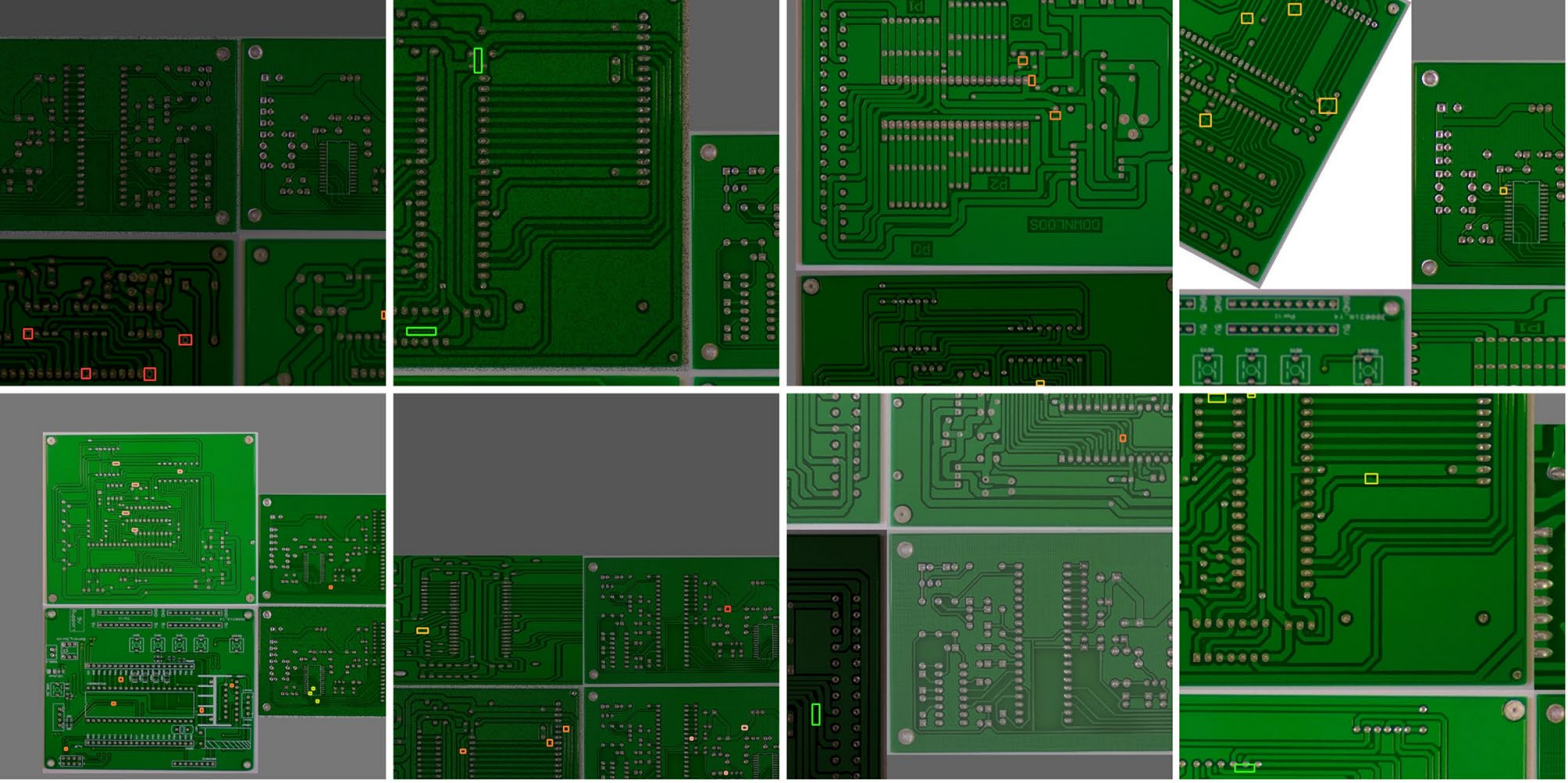
Example images of mosaic enhancement.

In order to prevent overfitting and enhance the generalization of the model, we have made reasonable adjustments to the data augmentation parameters during training. Data augmentation can generate additional training samples by modifying existing training data or creating synthetic data. We used classic methods such as image HSV enhancement, random scale sampling, random flip and mosaic enhancement. The Image augmentation hyperparameters are shown in Table 4. It is worth mentioning that when using mosaic enhancement, 4 images are read for each training, and a series of operations such as random scaling, flipping, cropping, and optical transformation are performed again, then these four images are stitched together, and the adjusted labels are passed into the network. This operation greatly enhances the diversity of training data and improves the model's ability to detect small targets. The example images mosaic enhancement is shown in Fig. 8.

**Table 4:** Augmentation hyperparameters.

Parameter	Value
HSV-Hue	0.015
HSV-saturation	0.7
HSV-value	0.4
Image scale	0.5
Image flip left–right	0.5
Mosaic	1.0

### Evaluation metrics

To evaluate the overall performance of the model, the evaluation metrics used in this experiment are precision (P), recall (R), average precision (AP), mean average precision (mAP), FPS, and model size. Their calculations are shown in Eqs. (1) – (5).

Precision represents the proportion of positive samples in the positive samples and is calculated as follows:

1 $$\mathrm{Precision}=\frac{\mathrm{TP}}{\mathrm{TP}+\mathrm{FP}}$$

Recall indicates the proportion of the predicted positive samples in the whole sample to the number of positive samples, and it is calculated as follows:

2 $$\mathrm{Recall}=\frac{\mathrm{TP}}{\mathrm{TP}+\mathrm{FN}}$$

The F1-Score represents the weighted average of precision and recall, and it is calculated as follows:

3 $$ {\text{F}}1 = \left( {\frac{2}{{ {\text{Recall}} ^{ - 1} + {\text{Precision}} ^{ - 1} }}} \right) = 2 \cdot \frac{{{\text{Precision}} \cdot {\text{ Recall}} }}{{ {\text{Precision}} + {\text{Recall}}}} $$

The formulas for calculating AP and mAP are as follows:

4 $$\mathrm{AP}={\int }_{0}^{1}\mathrm{P}(\mathrm{R})\mathrm{dR}$$

5 $$\mathrm{mAP}=\frac{{\Sigma }_{\mathrm{j}=1}^{\mathrm{S}}\mathrm{AP}\left(\mathrm{j}\right)}{\mathrm{S}}$$

In the above equation, S represents the number of all categories and is both the denominator and the sum of the AP of all categories.

### Experimental results and analysis

**Figure 9:**
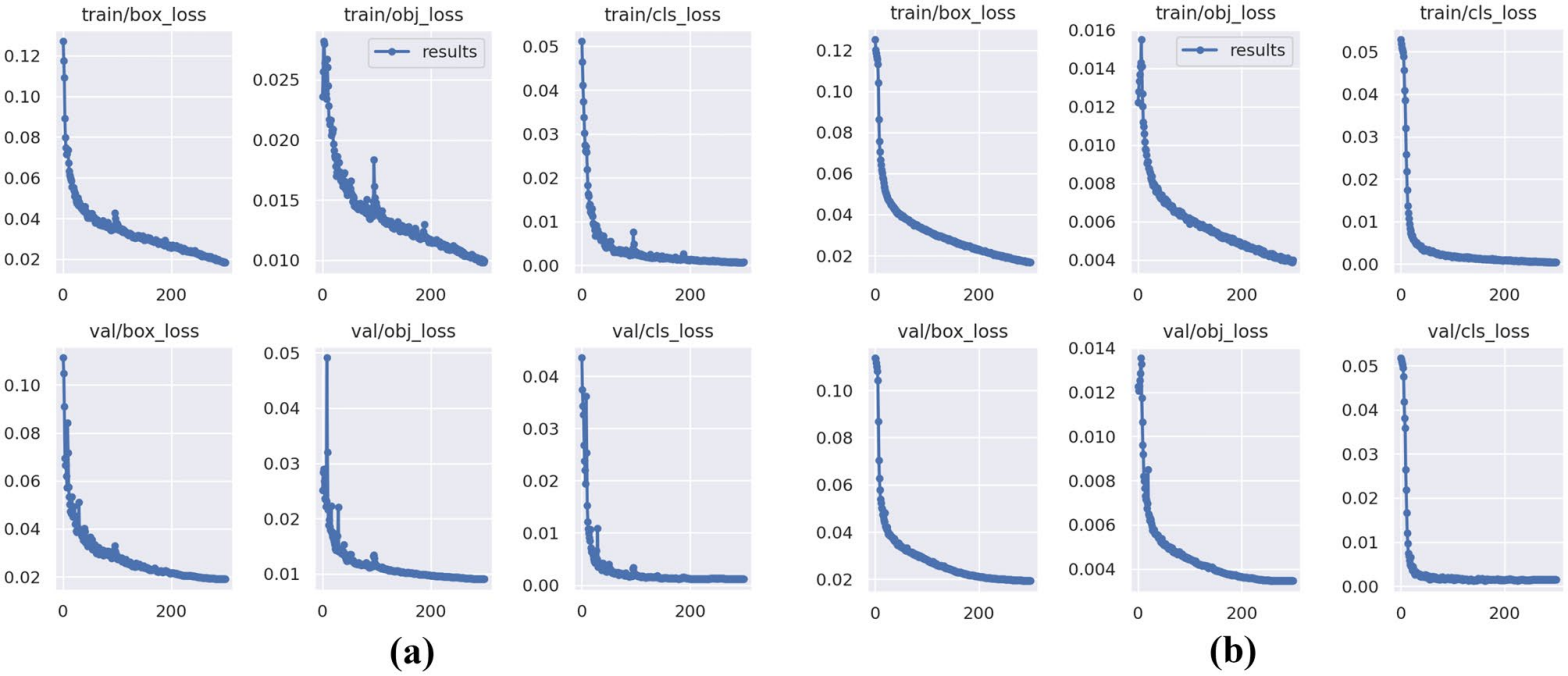
Training loss of YOLOv5 and GCC-YOLO: (**a**) Training loss of YOLOv5, (**b**) Training loss of GCC-YOLO.

**Figure 10:**
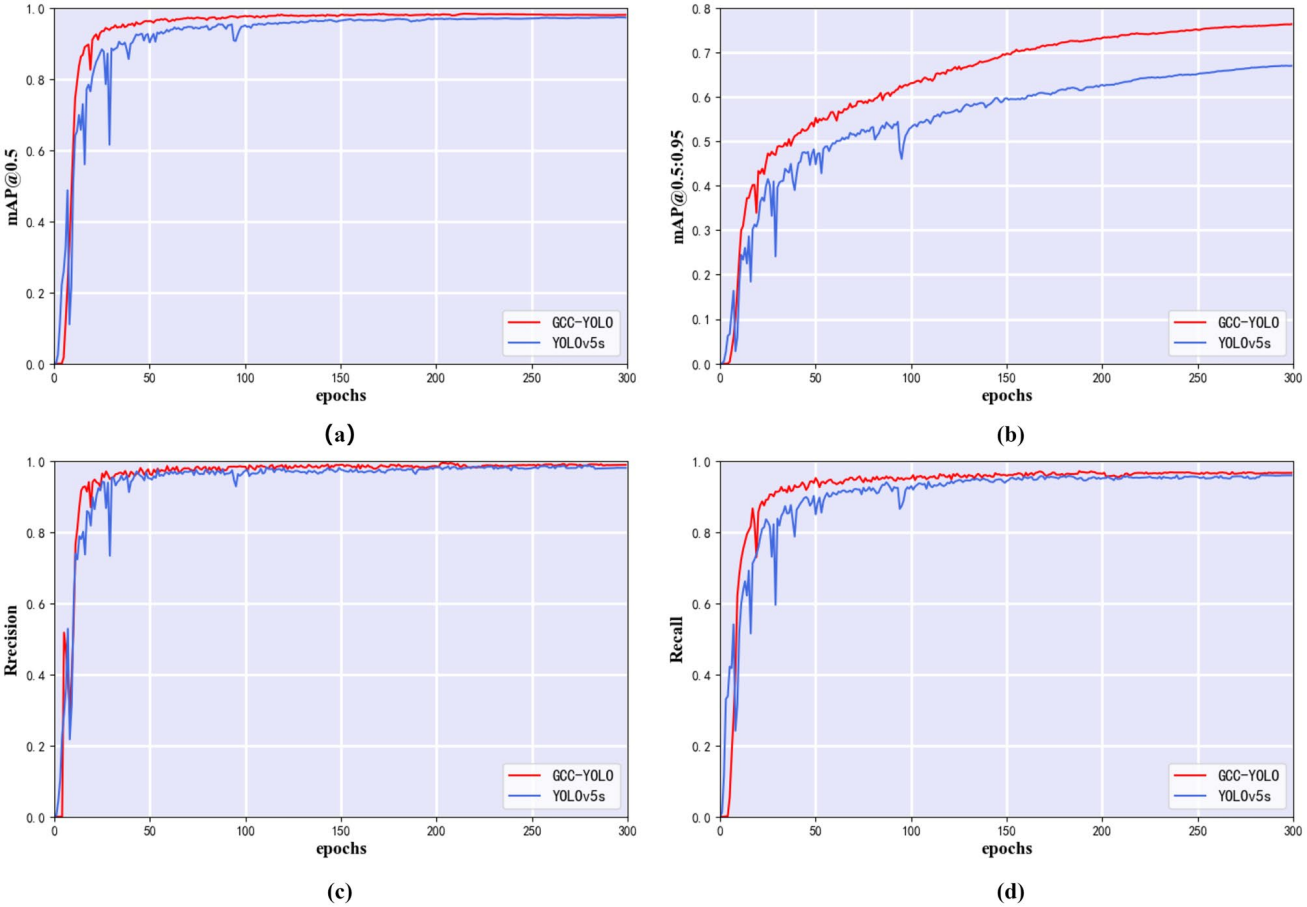
Comparison of training performance between YOLOv5 and GCC-YOLO, red lines indicate the GCC-YOLO and blur lines indicate the YOLOv5: (**a**) Training result of mAP@0.5, (**b**) Training result of mAP@0.5:0.95, (**c**) Training result of Precision, (**d**) Training result of Recall.

To verify the effectiveness of the various new modules proposed in this paper, an ablation experiment scheme is designed, as shown in Table 5. Scheme 1 is YOLOv5s; Scheme 2 is introducing the shallow high-resolution layer P2; Scheme 3 is adding the C3GC module in the backbone network and is based on Scheme 2; Scheme 4 is introducing the BiFPN feature fusion path and is based on Scheme 3; and Scheme 5 is adding the C3CM module as the detection head in the neck network and is based on Scheme 4. The ablation experiment results of the five schemes are shown in Table 6. Below, we discuss each observed improvement. In addition, we visualized the training loss and performance metrics of YOLOv5 and the improved GCC-YOLO, as shown in Figs. 9 and 10, respectively, to verify the performance and generalization performance of the improved model.

**Table 5:** Ablation experiment schemes.

Schemes	P2	C3GC	BiFPN	C3CM
1	×	×	×	×
2	√	×	×	×
3	√	√	×	×
4	√	√	√	×
5	√	√	√	√

**Table 6:** Ablation experiment result.

Schemes	AP (%)						Precision (%)	Recall (%)	mAP@0.5 (%)	mAP@0.5:0.95 (%)
	Mh	Mb	Oc	Sh	Sp	Sc				
1	99.5	97.5	98.5	99.4	95.8	95.8	98.8	95.5	97.7	68.4
2	99.5	96.9	98.3	99.4	96.3	95.7	98.1	96.5	97.7	73.3
3	99.5	98.6	98.4	99.4	97.1	95.6	99.1	96.4	98.1	75.2
4	99.5	99.0	98.5	99.3	97.0	96.0	99.1	96.3	98.2	75.6
5	99.5	98.3	98.6	99.2	97.3	96.1	99.0	97.3	98.2	76.7

#### Influence of high-resolution feature layer P2

According to the results of Scheme 2 in Table 6, after adding the P2 layer, the recall rate increased by 1% and mAP@0.5:0.95 increased by 4.9%. This indicates that by adding the P2 layer, the detailed information and localization information of shallow small targets can be extracted effectively, which helps to prevent missed detections.

#### Influence of improved backbone

The results of Scheme 3 in Table 6 show that by introducing global contextual attention enhancement to extract the C3GC module on the backbone network, the interference caused by shallow background noise can be effectively reduced, thus avoiding invalid feature propagation and making the model pay more attention to the effective area. Compared to Scheme 2, the introduction of C3GC resulted in a slight decrease in recall rate, while other indicators increased.

#### Influence of feature fusion

According to the results of Scheme 4 in Table 6, by introducing the BiFPN feature fusion path in the neck network, the shallow layer feature information of the jump connection backbone network is relieved, which reduces feature information loss when the shallow layer small target features are propagated in the network. Compared with Scheme 3, the detection precision for mouse bite and open circuit when there are many small targets is increased by 0.4% and 0.1%, respectively. However, we note that the fusion of too many shallow feature information also brings certain optimization problems, i.e., the detection precision of both the relatively large-sized short and spur defect type decreased by 0.1%, and the recall rate of the model decreased by 0.1%.

#### Influence of ConvMixer prediction heads

According to the results of Scheme 5 in Table 6, the introduction of the C3CM prediction head with a large field of view and pointwise convolution improves the recall rate and detection precision for small targets. Compared to Scheme 4, the model recall rate and mAP@0.5:0.95 increases by 1% and 1.1%, respectively.

#### Summary of model improvement performance

The loss function of YOLO model includes box loss, objectness loss, and classification loss. Box loss evaluates the algorithm's ability to accurately locate the center and boundary box of the object, objectness loss quantifies the likelihood of finding an object within a given region, and classification loss represents the accuracy of the algorithm in determining the correct category of the object. As shown in Fig. 9, GCC-YOLO has a faster convergence speed and a smoother loss curve compared to the baseline model YOLOv5, this indicates that GCC-YOLO achieved better performance during training and has better generalization ability. As shown in Fig. 10, compared with YOLOv5, GCC-YOLO has better performance in map@0.5, map@0.5:0.95, precision and recall, this indicates that the improved model can better detect small defects on the surface of PCB and has better detection accuracy.

### Comparison of algorithms

**Figure 11:**
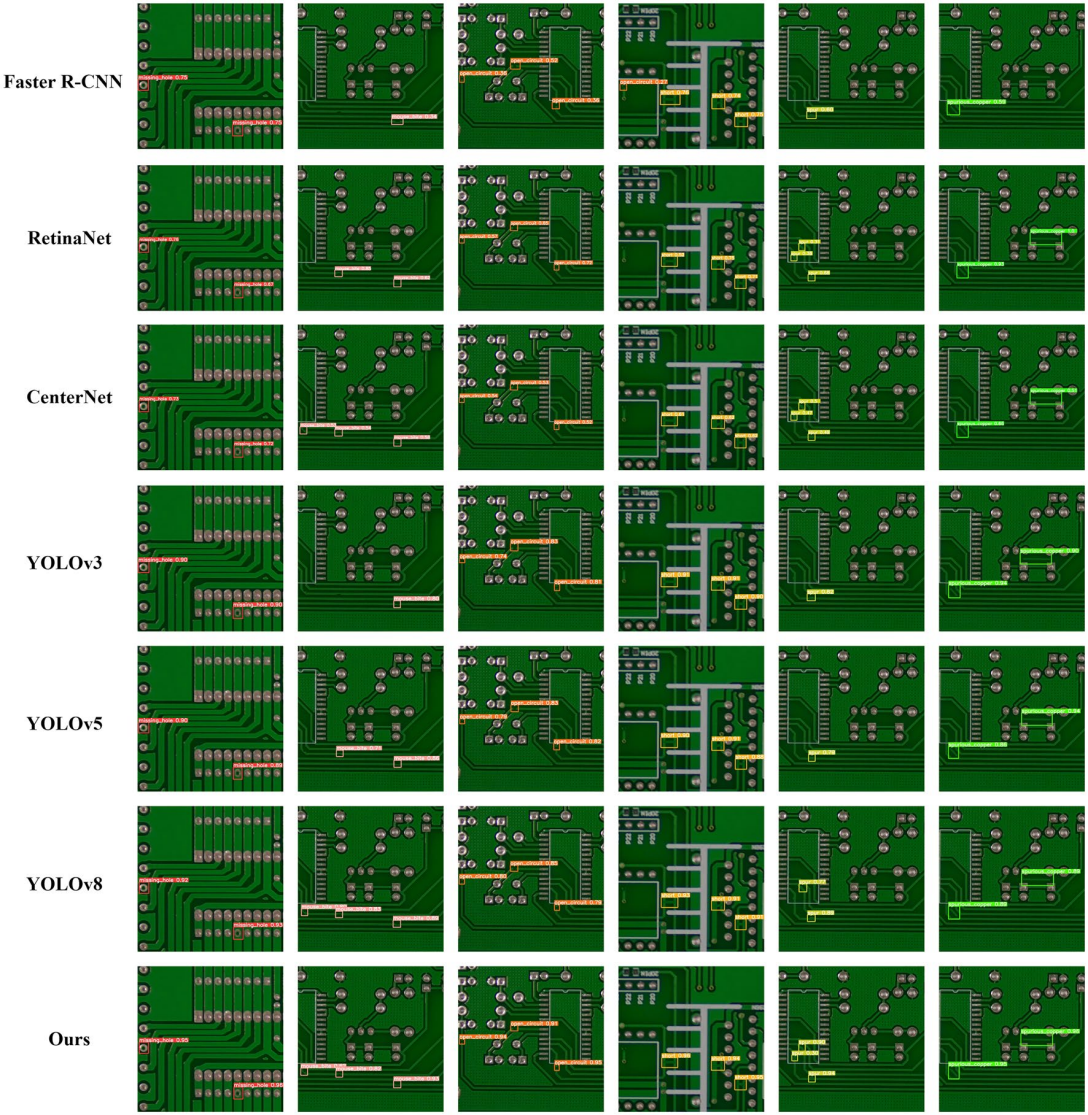
Detection results of advanced object detection algorithms.

To evaluate the detection performance of GCC-YOLO for tiny targets under complex texture interference, we first selected three advanced models^40–42^ that perform well in small object detection in the PCB dataset, as well as TPH-YOLO^37^ which has achieved outstanding results in small object detection in unmanned aerial vehicles, for comparative experiments. The experimental results of the five algorithms on the enhanced PCB dataset are shown in Table 7. Secondly, we compared the performance of GCC-YOLO with Faster R-CNN^8^, RetinaNet^15^, CenterNet^16^, YOLOv3^43^, YOLOv5, YOLOv8 and other adcanved target detection models, the comparison experimental data are shown in Table 8, and the detection results are shown in Fig. 11. In addition, we also compared the stability and complexity of GCC-YOLO with other YOLO models, and the results are shown in Table 9, because the lightweight model volume and lower model complexity are of great significance for the rapid deployment of PCB.

**Table 7:** Comparison of PCB defect detection algorithms and tiny target detection algorithms.

Models	AP (%)						Precision (%)	Recall (%)	mAP@0.5 (%)	mAP@0.5:0.95 (%)
	Mh	Mb	Oc	Sh	Sp	Sc				
Paper^40^	98.8	93.8	94.1	95.4	86.9	91.1	96.0	89.9	93.3	54.8
Paper^41^	99.5	97.3	98.7	98.7	96.6	94.4	99.1	95.8	97.5	75.4
Paper^42^	99.5	99.0	98.6	99.4	96.2	95.9	99.0	96.7	98.1	64.2
Paper^37^	99.5	98.3	98.4	99.0	95.9	95.1	97.9	96.8	97.7	66.6
Ours	99.5	98.3	98.6	99.2	97.3	96.1	99.0	97.3	98.2	76.7

**Table 8:** Comparison of advanced object detection algorithms.

Models	AP (%)						mAP@0.5 (%)	mAP@0.5:0.95 (%)	FPS (F/S)
	Mh	Mb	Oc	Sh	Sp	Sc			
Faster R-CNN^8^	97.5	89.2	94.4	94.8	82.9	88.2	91.2	43.8	12.0
RetinaNet^15^	90.7	90.0	87.5	90.5	88.3	85.8	88.8	52.9	19.7
CenterNet^16^	90.7	93.1	90.7	88.4	90.7	90.4	90.6	51.4	6.9
YOLOv3^43^	98.3	94.8	97.0	99.1	94.0	93.2	96.1	64.5	59.9
YOLOv5	99.5	97.5	98.5	99.4	95.8	95.8	97.7	68.4	89.3
YOLOv8	99.5	98.2	95.8	98.7	94.6	98.8	97.6	78.3	79.4
Ours	99.5	98.3	98.6	99.2	97.3	96.1	98.2	76.7	68.0

**Table 9:** Comparison of model complexity and stability of YOLO Series.

Model	Precision (%)	Recall (%)	F1-score (%)	Parameters	GFLOPS (G)	Volume (Mb)
YOLOv3^43^	97.3	92.7	94.9	61,524,355	154.6	117.0
YOLOv5	98.8	95.5	97.1	7,026,307	15.8	14.8
YOLOv8	98.2	95.4	96.8	11,127,906	28.4	21.4
Ours	99.0	97.3	98.1	6,874,836	17.8	14.5

#### Analysis between GCC-YOLO and other advanced PCB defect detection algorithms

According to the experimental results shown in Table 8, compared with other small target detection models, GCC-YOLO achieved the best Recall value of 97.3% in the enhanced PCB dataset, this shows that GCC-YOLO can effectively locate more small targets, and achieved the best detection accuracy in almost all types, except the mouse bite type, this demonstrates that GCC-YOLO has excellent performance in detecting small targets.

#### Analysis between GCC-YOLO and other advanced object detection algorithms

According to the detection results shown in Fig. 11, Faster R-CNN has the worst performance on the PCB dataset, with four missed detections and one false detection. The other algorithms also have missed detections, i.e., one for RetinaNet, four for YOLOv3 and YOLOv5s, three for YOLOv5s, and one for YOLOv8. These are mainly mouse bite and spur. Only GCC-YOLO correctly detects all the defect targets.

The comparison experiment results show that GCC-YOLO effectively improves the detection precision for various types of defects compared to several mainstream models. The AP values for missing hole, mouse bite and open circuit are 99.5%, 98.3%, and 98.6%, respectively; these are all better than those of the other algorithms. In terms of model performance, the mAP@0.5 and mAP@0.5:0.95 values of GCC-YOLO reach 98.2% and 76.7%, respectively. Compared to the original YOLOv5s, these are increases of 0.5% and 8.3%, respectively. Compared to the latest YOLOv8 algorithm, mAP@0.5 is increased by 0.6%. In terms of detection speed, GCC-YOLO achieves 68.0 FPS, comparing with Faster R-CNN 12.0 FPS, RetinaNet 12.9FPS, CenterNet 6.9 FPS, and YOLOv3 59.9 FPS; this makes GCC-YOLO more adaptable to industrial scenarios.

Model stability and complexity of YOLO Series are compared in Table 6. The detection precision, recall rate, and F1-Score of GCC-YOLO reach 99.0%, 97.3%, and 98.1%, respectively, outperforming the other algorithms. Thus, GCC-YOLO has the highest stability. The results of the complexity comparison experiment show that the parameters, GFLOPS, and model size of GCC-YOLO are 6,874,836, 17.8G, and 14.5 Mb, respectively. among which the parameters are the least compared with other networks, which is due to ConvMixer with depthwise convolution replacing the CSP structure in the original C3 module. GFLOPS is slightly increased in the proposed model compared to YOLOv5s but is lower than those of YOLOv3 and YOLOv8; hence, the improvements made in this paper do not significantly increase the complexity of the model. Finally, the model size is lower than 117 mb and 21.4 mb for YOLOv3 and YOLOv8, respectively, thus indicating that GCC-YOLO is a lightweight model suitable for the industrial needs of PCB lightweight detection.

## Conclusion

This paper proposes the GCC-YOLO model, which improves on the YOLOv5s network structure to address the challenge of detecting small targets on the PCB dataset. First, a shallow high-resolution P2 layer is introduced to avoid the problem of losing small target feature information due to excessive down-sampling. Second, introducing a feature extraction module with global contextual attention enhancement into the backbone network suppresses the background noise interference caused by shallow features and makes the model pay more attention to the target area. Then, introducing a BiFPN feature fusion path to replace the original PANet structure in the neck network reduces shallow feature loss due to the increase in model depth. Finally, introducing a lightweight prediction head with a large field of view further enhances the model's ability to detect small targets. Experimental results show that GCC-YOLO outperforms other mainstream object detection models on the PCB dataset in terms of overcoming the problems of false detection and missed detection. Furthermore, it has better detection precision and detection speed while also meeting the requirements of lightweight deployment. Therefore, it meets the industrial requirements of PCB surface defect detection.

In our current work, we have done a lot of design work for small target PCB surface defects with our model. However, the detection precision for the relatively large-sized spurious copper type defect has a lower precision value, and in each ablation experiment, it was at the relatively lowest detection accuracy. According to experimental data, the detection accuracy of spurious copper has continuously decreased after introducing the P2 layer and C3GC module. Large-scale experiments have shown that incorporating more large-scale feature map information is beneficial for detecting small targets, but does not provide any benefits for relatively large spurious copper defects in terms of size. And the fusion of shallow feature maps brings more noise interference. Meanwhile, the introduction of the C3GC module with anti-texture feature extraction ability may over-process these interferences, which affects the deeper feature information of the backbone network and thus affects the detection accuracy of relatively larger scale defects. Regarding the above issues, relevant studies have shown that improvements can be made from the following perspectives: (1) Introducing candidate box generation algorithms with adaptive adjustment can generate more fitting candidate boxes to improve the detection accuracy of a certain scale feature; (2) Introducing multi-scale feature fusion path with dynamic adjustment to balance the detection accuracy problem of different scales; (3) Introducing anchor-free mechanism and decoupling head network can also balance the problem of detection accuracy imbalance among different categories without generating candidate boxes and separating classification and positioning calculations. In the future, our subsequent work will continue the research from these three aspects.

## Data Availability

All data generated or analysed during this study are available in the Github repository. Links to the code and datasets are provided in the below hyperlinked text. Code and dataset of GCC-YOLO project: https://github.com/2462954048/GCC-YOLO-V2/tree/master.
